# Supplementary material for: Impact of organic compounds on the stability of influenza A virus in deposited 1-μL droplets
Source: mSphere. 2024 Aug 22;9(9):e00414-24. doi: 10.1128/msphere.00414-24 (PMC11423574; doi:10.1128/msphere.00414-24)
Supplement: Supplemental Information — Additional experimental details and supplemental figures and tables. [file msphere.00414-24-s0001.pdf]

Supplementary information for

Impact of Organic Compounds on the Stability of Influenza A Virus in deposited 1-µl droplets

Aline Schaub[1], Shannon C. David[1], Irina Glas[2], Liviana K. Klein[3], Kalliopi Violaki[4], Céline Terrettaz[1,4], Ghislain Motos[4], Nir Bluvshtein[3], Beiping Luo[3], Marie Pohl[2], Walter Hugentobler[4], Athanasios Nenes[4,5], Ulrich K. Krieger[3], Thomas Peter[3], Silke Stertz[2], Tamar Kohn[1]*

[1]Laboratory of Environmental Virology, School of Architecture, Civil & Environmental Engineering, École Polytechnique Fédérale de Lausanne, Lausanne, Switzerland

[2]Institute of Medical Virology, University of Zurich, Zurich, Switzerland

[3]Institute for Atmospheric and Climate Science, ETH Zurich, Zurich, Switzerland

[4]Laboratory of Atmospheric Processes and their Impacts, School of Architecture, Civil & Environmental Engineering, École Polytechnique Fédérale de Lausanne, Lausanne, Switzerland

[5]Center for The Study of Air Quality and Climate Change, Institute of Chemical Engineering Sciences, Foundation for Research and Technology Hellas, Patras, Greece

*Correspondence: tamar.kohn@epfl.ch

This PDF file includes:

12 pages

Supporting Methods

Figure S1 to S3

Tables S1 to S6

Supporting Methods

Analysis of lipids

Nasal mucus sample (50 µl) was mixed with 200 µl of $CHCl_3/CH_3OH$ 1:1 (v/v). The mixture was sonicated for 5 min, and then 200 µl of ammonium acetate 7.5 mM were added. After 5 min of centrifugation at 5000 x g, the lower organic phase was collected and evaporated under a stream of nitrogen. Total lipids were redissolved in 100 µl of acetonitrile and injected in an Agilent LC-ESI-Q-TOF/MS system equipped with an LC system (Agilent 1290 Infinity) coupled with a Q-TOF (Agilent 6530 Accurate-Mass). Chromatographic separation was performed with a Luna-HILIC (Phenomenex) column (100 mm, 2 mm I.D., 3 µm particle size). Gradient chromatographic separation was achieved by using H_2O as the mobile phase with 5 mM ammonium acetate (A) and acetonitrile with 5 mM ammonium acetate (B). The gradient elution was 1) 10% of (A) (and thus, 90% of (B)) from 0–2.5 min, 2) 50% (A) from 2.5–10 min, 3) 50% (A) from 10–12 min 4) 10% (A) from 12–14 min, and 5) 10% (A) from 14–17 min. The flow rate was set at 100 µl/min. The column temperature was set at 25 °C, and the injection volume was 2 µl. Ionization was performed in positive mode for all lipids, except for the fatty acids where negative ionization was applied. The following operational parameters were used: capillary voltage: 3500 V; drying gas: 8 l/min; nebulizer pressure: 35 psig; gas temperature: 350 °C; sheath gas temperature: 300 °C; sheath gas flow: 11 l/min; nozzle voltage: 1000 V; fragmentor voltage: 160 V; skimmer voltage: 65 V; and octopole RF: 750 V. The full scan MS data were recorded at a rate of 1 spectra s^{-1} in the range of m/z 50–1700 at 2 GHz. All the MS data were recorded with Agilent Mass Hunter Data Acquisition (B.05.00) and processed by Agilent Mass Hunter Qualitative Analysis (B.05.00). MS/MS experiments were carried out with a medium isolation width (∼4 amu) and a fixed collision energy of 45 eV for all the compounds. The instrument was calibrated daily before starting the analysis using the calibration solution provided by the manufacturer.

Analysis of main ions

Nasal mucus (200 µl) was diluted in 2 ml of ultrapure water (Milli-Q system, 18 MΩ.cm). The diluted solution was filtered through a 13-mm syringe filter (hydrophilic PTFE with 0.22-µm pore size). The filtered samples were injected in the ion chromatography (IC) after the addition of chloroform (10 µl). The main anions (Cl^-, NO_3^-, SO_4^{2-}, HPO_4^{2-}, $C_2O_4^{2-}$) were analyzed by IC after separation on a Dionex AS18 column (4x250 mm). The anions were determined with gradient elution at 1 ml/min with 23 mM KOH as eluent, and an ASRS-300 4 mm suppressor in auto suppression mode was used with applied current 90 mA. For the cations (Na^+, NH_4^+, K^+, Mg^{2+}, and Ca^{2+}), a CS12A-5µm (3x150 mm) column with a CSRS-300 4 mm suppressor was used. Separation was achieved under isocratic conditions with methane sulfonic acid (MSA) eluent (20 mM) and a flow rate of 0.5 ml/min. The detection limit ranged from 1 to 5 ppb for the main anions and cations and 3 ppb for phosphate ions.

Analysis of proteins

The total protein content was determined using a Qubit Protein Assay Quantification Kit (Life Technologies, Q33211).

Supporting Figures

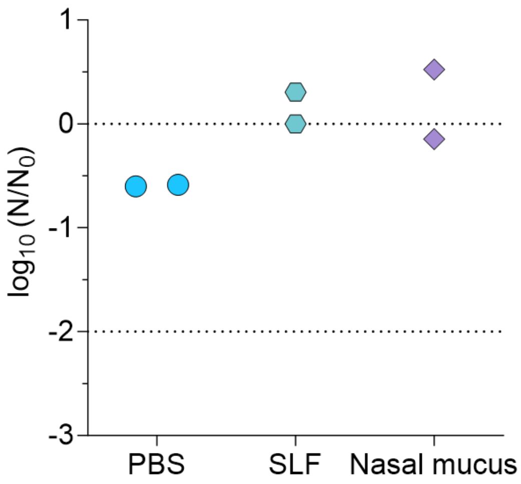

Figure S1: Control experiments conducted in bulk solutions for the three matrices studied (corresponding to droplet experiments shown in Figure 1 of the main text). Each data point represents one independent experiment. Minor inactivation was observed over the course of 1 h in PBS, but not in the other matrices.

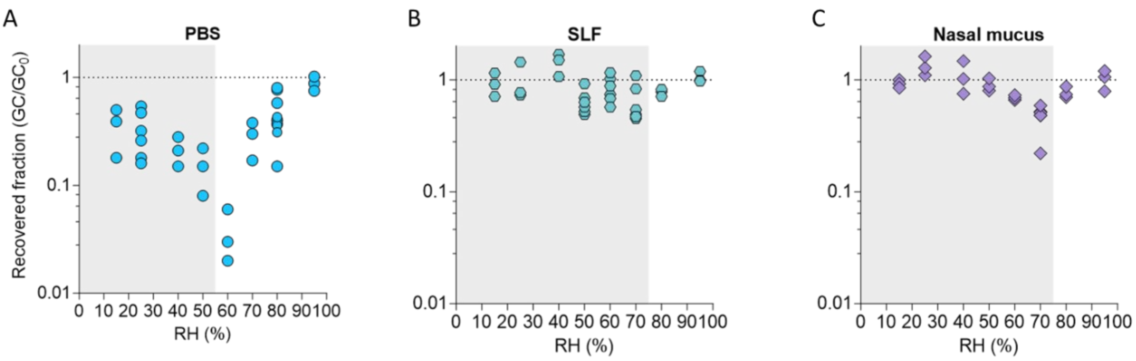

Figure S2: Recovered fraction of IAV genomic copies after 1 h at 15-95% RH in 1-µl droplets composed of (A) PBS, (B) SLF, and (C) nasal mucus. The recovery fraction (GC/GC_0) was determined from the ratio between the number of genomic copies in a droplet recovered after 1 h (GC) and the number of genomic copies in a droplet recovered immediately after deposition (GC_0).Each data point represents one individual droplet. Droplets in the gray shading were effloresced, whereas droplets outside of the shading were liquid.

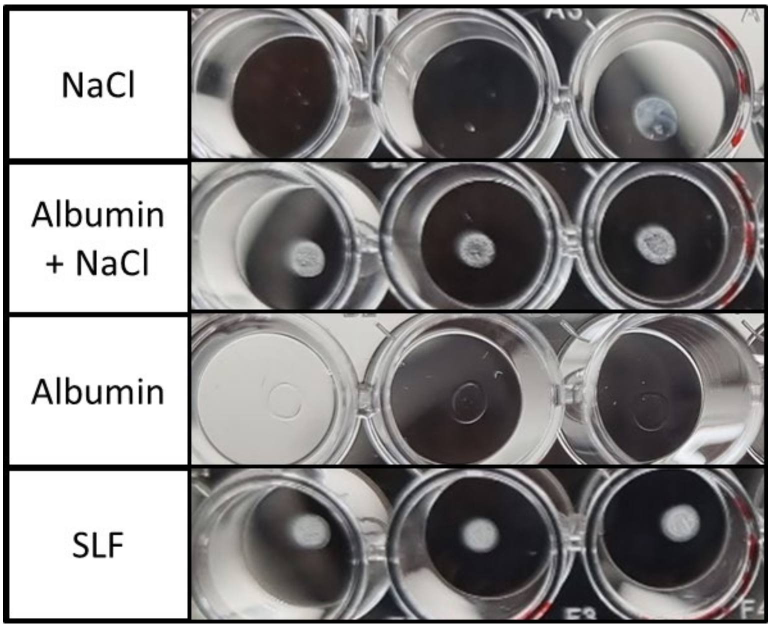

Figure S3: Droplet morphology after 1 h exposure at 60% RH. The initial NaCl and albumin concentrations are 8 and 8.8 g/l, respectively. The first two NaCl droplets are liquid, therefore barely distinguishable. Albumin:NaCl and SLF droplets are effloresced. Albumin droplets exhibit a “coffee-ring”.

Supporting Tables

Table S1: Check-list of experimental details as requested by MIQE guidelines according to Bustin, S. A. *et al.* The MIQE guidelines: Minimum information for publication of quantitative real-time PCR experiments. *Clin. Chem.* **55**, 611–622 (2009).

ITEM TO CHECK	PROVIDED Y/N	COMMENT
**EXPERIMENTAL DESIGN**		
Definition of experimental and control groups	Y	In materials and methods
Number within each group	Y	Specified in figures
**SAMPLE**		
Description	Y	In materials and methods
Microdissection or macrodissection	NA	
Processing procedure	Y	In materials and methods
If frozen - how and how quickly?	Y	In materials and methods
If fixed - with what, how quickly?	Y	In materials and methods
Sample storage conditions and duration (especially for FFPE samples)	Y	In materials and methods
**NUCLEIC ACID EXTRACTION**		
Procedure and/or instrumentation	Y	In materials and methods
Name of kit and details of any modifications	Y	In materials and methods
Details of DNase or RNAse treatment	Y	According to kit instructions
Contamination assessment (DNA or RNA)	Y	In materials and methods
Nucleic acid quantification	N	Not performed
Instrument and method	NA	
RNA integrity method/instrument	N	Not performed
RIN/RQI or Cq of 3' and 5' transcripts	NA	
Inhibition testing (Cq dilutions, spike or other)	Y	In materials and methods
**REVERSE TRANSCRIPTION**		
Complete reaction conditions	Y	In materials and methods
Amount of RNA and reaction volume	Y	In materials and methods
Priming oligonucleotide (if using GSP) and concentration	NA	
Reverse transcriptase and concentration	Y	According to kit instructions
Temperature and time	Y	In materials and methods
**qPCR TARGET INFORMATION**		
Sequence accession number	N	Not provided
Amplicon length	Y	In materials and methods
In silico specificity screen (BLAST, etc.)	NA	
Location of each primer by exon or intron (if applicable)	NA	
What splice variants are targeted?	NA	
**qPCR OLIGONUCLEOTIDES**		
Primer sequences	Y	In materials and methods
Probe sequences	Y	In materials and methods
Location and identity of any modifications	NA	
**qPCR PROTOCOL**		
Complete reaction conditions	Y	In materials and methods
Reaction volume and amount of cDNA/DNA	Y	In materials and methods
Primer, (probe), Mg++ and dNTP concentrations	Y	According to kit instructions
Polymerase identity and concentration	Y	According to kit instructions
Buffer/kit identity and manufacturer	Y	In materials and methods
Additives (SYBR Green I, DMSO, etc.)	Y	According to kit instructions
Complete thermocycling parameters	Y	In materials and methods
Manufacturer of qPCR instrument	Y	In materials and methods
**qPCR VALIDATION**		
Specificity (gel, sequence, melt, or digest)	N	Performed but not reported
For SYBR Green I, Cq of the NTC	N	Performed but not reported
Standard curves with slope and y-intercept	Y	In materials and methods
PCR efficiency calculated from slope	Y	In materials and methods
r2 of standard curve	Y	In materials and methods
Linear dynamic range	Y	In materials and methods
Cq variation at lower limit	Y	In materials and methods
Evidence for limit of detection	Y	In materials and methods
**DATA ANALYSIS**		
qPCR analysis program (source, version)	Y	In materials and methods
Cq method determination	Y	In materials and methods
Outlier identification and disposition	NA	
Results of NTCs	N	Performed but not reported
Justification of number and choice of reference genes	NA	
Description of normalization method	NA	
Number and concordance of biological replicates	Y	Specified in figures
Number and stage (RT or qPCR) of technical replicates	Y	In materials and methods
Repeatability (intra-assay variation)	N	Not performed
Statistical methods for result significance	Y	In materials and methods
Software (source, version)	Y	In materials and methods

Table S2: The ions composition of nasal mucus. Note that the concentrations are given in mg/l.

Ions	Concentration (mg/l)
Sodium (Na^+)	646.014
Ammonium (NH_4^+)	15.640
Potassium (K^+)	38.815
Magnesium (Mg^{2+})	2.898
7.24	7.241
Chloride (Cl^-)	34.201
Nitrate (NO_3^-)	13.352
Sulfate (SO_4^{2-})	0.828
Phosphates (PO_4^{3-})	1.456
**Total (mg/l)**	**760.444**

Table S3: The lipids composition of nasal mucus. Note that the concentrations are given in mg/l. The total lipid concentration is 112.322 mg/l.

**Phosphatidyl-cholines (PC)**	**Concentration (mg/l)**	**Phosphatidyl-ethanolamine (PE)**	**Concentration (mg/l)**	**Fatty acids (C)**	**Concentration (mg/l)**	**Sphingomyelins (SM)**	**Concentration (mg/l)**
PC(16:0/18:1)	0.990	PE(16:0/18:1)	5.298	C16:0	23.734	SM(d34:1)	29.434
PC(18:1/18:1)	0.961	PE(18:1/18:1)	2.247	C18:1	2.381	SM(d42:2)	10.504
PC(16:0/16:1)	0.738	PE(16:0/20:5)	2.055	C14:0	0.757	SM(d34:0)	5.273
PC(18:0/18:1)	0.735	PE(18:0/20:3)	2.032	C18:2	0.399	SM(d32:1)	2.761
PC(16:0/16:0)	0.525	PE(18:0/20:4)	1.702	C20:4	0.399	SM(d42:1)	2.481
PC(16:0/18:2)	0.457	PE(16:0/18:1)	1.325	C16:1	0.385	SM(d36:1)	1.631
PC(16:0/20:4)	0.453	PE(18:0/18:1)	1.321	C20:0	0.370	SM(d40:1)	1.299
PC(18:1/20:4)	0.383	PE(16:0/20:4)	0.723	C20:3	0.310	SM(d40:2)	1.147
PC(14:0/16:0)	0.381	PE(16:0/22:6)	0.510	C15:0	0.278	SM(d34:2)	0.858
PC(16:0/20:3)	0.376	PE(18:0/22:6)	0.336	C20:2	0.153	SM(d38:1)	0.759
PC(18:0/20:3)	0.352	PE(18:0/22:6)	0.336	C24:0	0.142	SM(d42:3)	0.405
PC(18:0/20:4)	0.328	PE(18:0/22:4)	0.257	C20:1	0.132	SM(d35:1)	0.295
		PE(18:0/22:4)	0.257	C17:1	0.132		
		PE(18:1/18:2)	0.240	C22:0	0.124		
		PE(16:0/20:3)	0.240				
		PE(16:0/20:4)	0.223				
**Total (g/l)**	**6.680**		**19.102**		**29.695**		**56.845**

Table S4: Two-way ANOVA with Tukey's multiple comparisons test for data shown in Figure 1. Number of families = 3, number of comparisons per family = 3, alpha = 0.05.

Tukey's multiple comparisons test	Mean Diff.	95.00% CI of diff.	Below threshold?	Summary	Adjusted p-value
PBS					
15-40% RH vs. 50-70% RH	0.2746	-0.07092 to 0.6200	No	ns	0.146
15-40% RH vs. 80-95% RH	-1.201	-1.520 to -0.8807	Yes	****	<0.0001
50-70% RH vs. 80-95% RH	-1.475	-1.821 to -1.130	Yes	****	<0.0001
SLF					
15-40% RH vs. 50-70% RH	-0.4583	-0.7781 to -0.1384	Yes	**	0.0028
15-40% RH vs. 80-95% RH	-0.9649	-1.378 to -0.5519	Yes	****	<0.0001
50-70% RH vs. 80-95% RH	-0.5066	-0.8759 to -0.1373	Yes	**	0.0044
Nasal mucus					
15-40% RH vs. 50-70% RH	0.6311	0.2617 to 1.000	Yes	***	0.0003
15-40% RH vs. 80-95% RH	0.4506	0.03767 to 0.8635	Yes	*	0.0291
50-70% RH vs. 80-95% RH	-0.1805	-0.5934 to 0.2324	No	ns	0.5518

Table S5: Ordinary one-way ANOVA with Tukey's multiple comparisons test for data shown in Figure 3. Number of families = 1, number of comparisons per family = 10, alpha = 0.05.

Tukey's multiple comparisons test	Mean Diff.	95.00% CI of diff.	Below threshold?	Summary	Adjusted p-value
No protein vs. No lipid	-0.9491	-1.821 to -0.07715	Yes	*	0.0285
No protein vs. No antioxidant	-0.7867	-1.659 to 0.08526	No	ns	0.0903
No protein vs. Only HBSS	0.9409	0.06893 to 1.813	Yes	*	0.0303
No protein vs. SLF	-1.434	-2.110 to -0.7591	Yes	****	<0.0001
No lipid vs. No antioxidant	0.1624	-0.7095 to 1.034	No	ns	0.9805
No lipid vs. Only HBSS	1.89	1.018 to 2.762	Yes	****	<0.0001
No lipid vs. SLF	-0.4854	-1.161 to 0.1900	No	ns	0.2423
No antioxidant vs. Only HBSS	1.728	0.8556 to 2.599	Yes	****	<0.0001
No antioxidant vs. SLF	-0.6478	-1.323 to 0.02761	No	ns	0.0643
Only HBSS vs. SLF	-2.375	-3.051 to -1.700	Yes	****	<0.0001

Table S6: Ordinary one-way ANOVA with Tukey's multiple comparisons test for data shown in Figure 4. Number of families = 1, number of comparisons per family = 78, alpha = 0.05.

Tukey's multiple comparisons test	Mean Diff.	95.00% CI of diff.	Below threshold?	Summary	Adjusted p-value
NaCl vs. 10^{-10}:1	-0.1338	-1.915 to 1.648	No	ns	>0.9999
NaCl vs. 10^{-9}:1	-0.184	-1.965 to 1.598	No	ns	>0.9999
NaCl vs. 10^{-8}:1	0.204	-1.577 to 1.986	No	ns	>0.9999
NaCl vs. 10^{-7}:1	0.2013	-1.179 to 1.581	No	ns	>0.9999
NaCl vs. 10^{-5}:1	-0.3092	-2.091 to 1.472	No	ns	0.9998
NaCl vs. 10^{-3}:1	-0.4036	-2.185 to 1.378	No	ns	0.9979
NaCl vs. 0.55:1	-0.8072	-2.589 to 0.9743	No	ns	0.7877
NaCl vs. 0.11:1	-0.9406	-2.321 to 0.4394	No	ns	0.3161
NaCl vs. 1.1:1	-0.841	-2.221 to 0.5390	No	ns	0.4485
NaCl vs. 2.1:1	-1.972	-3.753 to -0.1904	Yes	*	0.0261
NaCl vs. Albumin	-1.764	-3.144 to -0.3840	Yes	**	0.0093
NaCl vs. SLF	-1.64	-2.709 to -0.5714	Yes	**	0.0022
10^{-10}:1 vs. 10^{-9}:1	-0.05012	-2.304 to 2.203	No	ns	>0.9999
10^{-10}:1 vs. 10^{-8}:1	0.3379	-1.916 to 2.591	No	ns	>0.9999
10^{-10}:1 vs. 10^{-7}:1	0.3352	-1.616 to 2.287	No	ns	0.9998
10^{-10}:1 vs. 10^{-5}:1	-0.1753	-2.429 to 2.078	No	ns	>0.9999
10^{-10}:1 vs. 10^{-3}:1	-0.2698	-2.523 to 1.984	No	ns	>0.9999
10^{-10}:1 vs. 0.55:1	-0.6734	-2.927 to 1.580	No	ns	0.9807
10^{-10}:1 vs. 0.11:1	-0.8067	-2.758 to 1.145	No	ns	0.8607
10^{-10}:1 vs. 1.1:1	-0.7071	-2.659 to 1.244	No	ns	0.9317
10^{-10}:1 vs. 2.1:1	-1.838	-4.092 to 0.4154	No	ns	0.151
10^{-10}:1 vs. Albumin	-1.63	-3.582 to 0.3215	No	ns	0.1346
10^{-10}:1 vs. SLF	-1.506	-3.252 to 0.2390	No	ns	0.1143
10^{-9}:1 vs. 10^{-8}:1	0.388	-1.865 to 2.641	No	ns	0.9998
10^{-9}:1 vs. 10^{-7}:1	0.3853	-1.566 to 2.337	No	ns	0.9994
10^{-9}:1 vs. 10^{-5}:1	-0.1252	-2.379 to 2.128	No	ns	>0.9999
10^{-9}:1 vs. 10^{-3}:1	-0.2197	-2.473 to 2.034	No	ns	>0.9999
10^{-9}:1 vs. 0.55:1	-0.6233	-2.877 to 1.630	No	ns	0.9891
10^{-9}:1 vs. 0.11:1	-0.7566	-2.708 to 1.195	No	ns	0.9
10^{-9}:1 vs. 1.1:1	-0.657	-2.609 to 1.295	No	ns	0.9565
10^{-9}:1 vs. 2.1:1	-1.788	-4.041 to 0.4655	No	ns	0.1716
10^{-9}:1 vs. Albumin	-1.58	-3.532 to 0.3716	No	ns	0.1564
10^{-9}:1 vs. SLF	-1.456	-3.202 to 0.2892	No	ns	0.1354
10^{-8}:1 vs. 10^{-7}:1	-0.002699	-1.954 to 1.949	No	ns	>0.9999
10^{-8}:1 vs. 10^{-5}:1	-0.5132	-2.767 to 1.740	No	ns	0.9978
10^{-8}:1 vs. 10^{-3}:1	-0.6077	-2.861 to 1.646	No	ns	0.9911
10^{-8}:1 vs. 0.55:1	-1.011	-3.265 to 1.242	No	ns	0.7963
10^{-8}:1 vs. 0.11:1	-1.145	-3.096 to 0.8070	No	ns	0.4962
10^{-8}:1 vs. 1.1:1	-1.045	-2.997 to 0.9065	No	ns	0.6087
10^{-8}:1 vs. 2.1:1	-2.176	-4.429 to 0.07753	No	ns	0.0616

10^{-8}:1 vs. Albumin	-1.968	-3.920 to -0.01642	Yes	*	0.0475
10^{-8}:1 vs. SLF	-1.844	-3.590 to -0.09884	Yes	*	0.0354
10^{-7}:1 vs. 10^{-5}:1	-0.5105	-2.462 to 1.441	No	ns	0.993
10^{-7}:1 vs. 10^{-3}:1	-0.605	-2.557 to 1.347	No	ns	0.9749
10^{-7}:1 vs. 0.55:1	-1.009	-2.960 to 0.9430	No	ns	0.6507
10^{-7}:1 vs. 0.11:1	-1.142	-2.735 to 0.4515	No	ns	0.263
10^{-7}:1 vs. 1.1:1	-1.042	-2.636 to 0.5511	No	ns	0.3629
10^{-7}:1 vs. 2.1:1	-2.173	-4.125 to -0.2217	Yes	*	0.025
10^{-7}:1 vs. Albumin	-1.965	-3.559 to -0.3718	Yes	*	0.0122
10^{-7}:1 vs. SLF	-1.842	-3.175 to -0.5085	Yes	**	0.0051
10^{-5}:1 vs. 10^{-3}:1	-0.09448	-2.348 to 2.159	No	ns	>0.9999
10^{-5}:1 vs. 0.55:1	-0.498	-2.752 to 1.755	No	ns	0.9983
10^{-5}:1 vs. 0.11:1	-0.6314	-2.583 to 1.320	No	ns	0.9665
10^{-5}:1 vs. 1.1:1	-0.5318	-2.483 to 1.420	No	ns	0.9903
10^{-5}:1 vs. 2.1:1	-1.663	-3.916 to 0.5907	No	ns	0.2344
10^{-5}:1 vs. Albumin	-1.455	-3.406 to 0.4968	No	ns	0.2248
10^{-5}:1 vs. SLF	-1.331	-3.077 to 0.4144	No	ns	0.2044
10^{-3}:1 vs. 0.55:1	-0.4036	-2.657 to 1.850	No	ns	0.9998
10^{-3}:1 vs. 0.11:1	-0.5369	-2.488 to 1.415	No	ns	0.9896
10^{-3}:1 vs. 1.1:1	-0.4373	-2.389 to 1.514	No	ns	0.9981
10^{-3}:1 vs. 2.1:1	-1.568	-3.822 to 0.6852	No	ns	0.2935
10^{-3}:1 vs. Albumin	-1.36	-3.312 to 0.5913	No	ns	0.2918
10^{-3}:1 vs. SLF	-1.237	-2.982 to 0.5089	No	ns	0.2747
0.55:1 vs. 0.11:1	-0.1333	-2.085 to 1.818	No	ns	>0.9999
0.55:1 vs. 1.1:1	-0.03377	-1.985 to 1.918	No	ns	>0.9999
0.55:1 vs. 2.1:1	-1.165	-3.418 to 1.089	No	ns	0.6506
0.55:1 vs. Albumin	-0.9567	-2.908 to 0.9948	No	ns	0.7097
0.55:1 vs. SLF	-0.8331	-2.579 to 0.9124	No	ns	0.7377
0.11:1 vs. 1.1:1	0.09957	-1.494 to 1.693	No	ns	>0.9999
0.11:1 vs. 2.1:1	-1.031	-2.983 to 0.9202	No	ns	0.6244
0.11:1 vs. Albumin	-0.8234	-2.417 to 0.7701	No	ns	0.6508
0.11:1 vs. SLF	-0.6998	-2.033 to 0.6334	No	ns	0.6325
1.1:1 vs. 2.1:1	-1.131	-3.082 to 0.8207	No	ns	0.5113
1.1:1 vs. Albumin	-0.923	-2.516 to 0.6705	No	ns	0.5119
1.1:1 vs. SLF	-0.7993	-2.133 to 0.5338	No	ns	0.4687
2.1:1 vs. Albumin	0.208	-1.744 to 2.160	No	ns	>0.9999
2.1:1 vs. SLF	0.3316	-1.414 to 2.077	No	ns	0.9996
Albumin vs. SLF	0.1236	-1.210 to 1.457	No	ns	>0.9999
